# Are Pregnant Women with Chronic Helminth Infections More Susceptible to Congenital Infections?

**DOI:** 10.3389/fimmu.2014.00053

**Published:** 2014-02-12

**Authors:** Amir Abdoli, Majid Pirestani

**Affiliations:** ^1^Department of Parasitology, Faculty of Medical Sciences, Kashan University of Medical Science, Kashan, Iran; ^2^Department of Parasitology, Faculty of Medical Sciences, Tarbiat Modares University, Tehran, Iran

**Keywords:** pregnancy, helminth infections, congenital infections, immune response, intracellular pathogens, T-helper 1, T-helper 2, immunoregulation

## Some Similarities between Immune Response during Pregnancy and Chronic Helminth Infections Exist

Pregnancy is a unique immunological status with different hormonal and immunological alterations. Pregnancy hormones (including progesterone, glucocorticoids, estradiol, and estriol) increase over the course of pregnancy and significantly modulate the immunological shift that occurs over the three trimesters of pregnancy (1). In contrast, helminth infections are the most common infectious diseases in developing countries. Soil-transmitted helminths (ascariasis, trichuriasis, and hookworm), schistosomiasis, and lymphatic filariasis are the most common helminthiasis. Most helminth infections have minor clinical symptoms; thus, the infection is left untreated and may remain chronic for multiple years (2).

It is well-documented that a woman’s immune response during pregnancy and chronic helminth infections shift toward Type 2 immunity and anti-inflammatory cytokines (1, 3–6). During pregnancy, the activities of CD4+ T cells into helper T cell type 2 (Th2) and their anti-inflammatory cytokines [including interleukin-4 (IL-4), IL-5, and IL-10] increase. Inversely, the activities of T-helper 1 (Th1) cells and their inflammatory cytokines [as well as inflammatory macrophages and natural killer (NK) cells] decrease (1). These immunological shifts away from inflammatory responses are necessary for a successful pregnancy (1, 3). For example, activating cytotoxic cells (including NK or T cells and inflammatory cytokines such as IFN-γ and TNF-α) can damage the placenta and fetus (3). Moreover, the elevation of anti-inflammatory factors like IL-10 can prevent spontaneous abortions in murine models (7).

Similar to immune response during pregnancy, the immune response during a helminth infection shifts toward Th2 and anti-inflammatory status. During chronic helminth infections, the increased activation and expansion of CD4+ Th2 cells (including eosinophils, mast cells, basophils, and the antibody isotypes IgG1, IgG4, and IgE) occur. Moreover, production of the cytokines IL-4, IL-5, IL-9, IL-10, IL-13, and IL-21 and transforming growth factor-β (TGF-β) increases (4, 5). On the other hand, several studies in murine models reveal that helminth infections manipulate host hormones for its own benefit [reviewed in Ref. (8)]. In the case of *Taenia crassiceps* infection (a model for human cysticercosis), infected female mice showed an increase estrogen levels after 8 weeks post-infection. Infected male also showed increase serum estradiol and decrease testosterone levels. These hormonal changes lead to increase parasite density in both genders (9). Progesterone treatment in mice with *T. crassiceps* infection increased parasite loads and expression of the Th2 cytokine profile (IL-4, IL-6, and IL-10) in both genders (10). These findings reveal that female sex hormones are favorable for *T. crassiceps* infection and the infection is associated with Type 2 immunity. Hormonal regulation during helminth infections is often associated with increased parasite survival and Type 2 immunity [reviewed in Ref. (11, 12)], which negatively affect immune response to intracellular pathogens.

In contrast to immune response during pregnancy and chronic helminth infections, the protective immune response against the majority of the intracellular pathogens are mediated by Th1 cells and their cytokines (including IFN-γ, TNF-α, and IL-1) (13). Furthermore, increased activation and expansion of cytotoxic CD8+ T cells, NK cells, neutrophils, and macrophages occur during infection with intracellular pathogens (4, 13).

Therefore, immune system during pregnancy or helminth infections gives a weaker response to the infections that require strong Th1 immune response. So, it is plausible that a pregnant woman with chronic helminth infections is more susceptible to acquiring congenital infections due to synergic immunoregulatory effects of pregnancy and chronic helminth infections (see the next sections).

## Increase in Susceptibility and Severity to Infectious Diseases during Pregnancy

Immunological shifts toward Th2 response during pregnancy increases a woman’s susceptibility and severity to several infectious diseases such as malaria, measles, influenza, and toxoplasmosis (1, 14, 15). The results of a recent systematic review revealed that pregnant women are more susceptible to the acquisition of malaria, HIV infection, and listeriosis. Also, pregnancy increased the severity of malaria, influenza, hepatitis E, herpes simplex virus (HSV), measles, and smallpox (14). Thus, infectious diseases in pregnant women are more common and severe due to physiological immunoregulation that require for a successful pregnancy.

## Increases in Susceptibility and Severity to Infectious Diseases during Chronic Helminth Infections

Activation of Th2 immune responses and immunoregulatory pathways during helminth infections potentially downregulates the effector functions that mediate type 1 immune responses during intracellular pathogens; therefore, both the severity of and susceptibility to intracellular pathogens are increasing (16). Several studies support the finding that helminth infections modulate immune responses to HIV infection (16), *Mycobacterium tuberculosis* (16–18) and *Plasmodium* species (16, 19, 20). Eradication of helminth infections in HIV-positive patients significantly inhibits the progression of HIV infection by attenuation of the plasma viral load and increasing CD4 counts (21). The results of a randomized, double blind, placebo-controlled trial demonstrated that treatment of *Ascaris lumbricoides* infection in HIV-positive patients significantly increased CD4+ T cell counts and decreased viral load compared with placebo (22). Moreover, albendazole-treated patients had significantly lower plasma IL-10 levels (23). A study among Tanzanian women of reproductive age showed that the prevalence of HIV in women with urogenital schistosomiasis was threefold higher than women without urogenital schistosomiasis (24). Furthermore, it was observed that the densities of CD4+ T cells and macrophages in genital mucosa surrounding *Schistosoma haematobium* eggs were significantly higher than in cervicovaginal mucosa without ova (*p* = 0.034 and *p* = 0.018, respectively) (25). In the subject of helminth–tuberculosis co-infections, Elias et al. (26) observed that individuals with helminth infections had significantly lower levels of IFN-γ production to *M. tuberculosis* antigens compared with the albendazole-treated group. Helminth co-infections also impaired efficient host immune responses to different bacterial pathogens (27–32) as well as to *Leishmania* spp (33, 34) and *Toxoplasma gondii* (35). Therefore, immunoregulation during chronic helminth infections provides a suitable circumstance for acquisition of several infectious diseases.

## Increased Risk of Congenital Infections in Pregnant Women with Chronic Helminth Infections

As mentioned above, both pregnancy and chronic helminth infections increase susceptibility and severity toward the intracellular pathogens due to downregulation of type 1 immune response. This condition raises an important question: do the pregnancy and chronic helminth infections have synergic immunoregulatory effects? If this hypothesis is correct, pregnant women with chronic helminth infections may be more susceptible to congenital infections. However, there are some studies conducted in this regard (Table S1 in Supplementary Material). As reported by Gallagher et al. (36), helminth infections increase the risk of mother-to-child transmission of HIV infection. HIV-positive pregnant mothers with chronic helminth co-infection were found to have sevenfold greater risk of HIV transmission to their offspring (OR = 7.3, 95% CL 2.4–33.7). Moreover, the increased risk of HIV transmission was significantly associated with higher production of IL-5/IL-13 by cord blood lymphocytes (*p* < 0.001) (36). The results from a randomized, placebo-controlled trial among HIV-positive pregnant women in Uganda showed that hookworm and *Trichuris* infections were significantly associated with increased HIV viral load during pregnancy. Moreover, when treated with albendazole, some experienced reduced viral load within 6 weeks after treatment (37). The results of a cross-sectional study in HIV-positive pregnant women in Rwanda revealed that the prevalence of soil-transmitted helminths was 38%, malaria was 21%, and malaria–helminth co-infection was 10%. Also, helminth infections were associated with lower levels of hemoglobin and CD4 counts in HIV-positive mothers (38).

It has been proven the efficient vaccine against intracellular infections requiring cellular immunity and Th1 components (39). There is also evidence that maternal helminth infections suppress immune response to postnatal immunization (40). Elliott et al. (41) observed that maternal hookworm infection was associated with reduced maternal IFN-γ responses to crude culture filtrate proteins (CFP) of *M. tuberculosis*. Interestingly, an increase IFN-γ, in response to CFP, was found in mothers who were treated with albendazole, but not in untreated mothers. Conversely, lower levels of IFN-γ, in response to CFP, was observed in infants of hookworm-infected mothers who were treated with albendazole compared to those mothers receiving a placebo (41). It was also observed that the offspring of mothers infected with a filarial worm (*Mansonella perstans*) had higher levels of IL-10, in responses to CFP, and tetanus toxoid (42). Webb et al. (43) observed that albendazole therapy during pregnancy was associated with lower levels of IL-5 and IL-13 responses to tetanus toxoid in infants of mothers with hookworm infection (see details in Table S1 in Supplementary Material) (43).

Other studies were conducted in the area of malaria–helminth co-infection during pregnancy (20, 44–48). A recent meta-analysis by Naing et al. (20) revealed that pregnant women with hookworm infection had 1.36 times (OR: 1.36; 95% CI: 1.17–1.59) higher risk of malaria infection than those mothers without hookworm infection. Moreover, the risk of malaria in primigravid mothers with soil-transmitted helminth infections was 1.6 times (OR: 1.61; 95% CI: 1.3–1.99) higher than multigravid mothers (20). The results of a cross-sectional study among pregnant women in Ghana showed that mothers with intestinal helminth infections were at 4.8 times higher risk of acquiring *Plasmodium falciparum* infection (47). A study among pregnant women in Uganda demonstrated that women with hookworm and *M. perstans* infections had higher risk of *P. falciparum* infection (44). The odds ratio for *P. falciparum* infection in mothers infected with hookworm was 1.53 (CI, 1.09–2.14), for mothers infected with *M. perstans* 2.33 (CI, 1.47–3.69) and for mothers infected with both hookworm and *M. perstans* 1.85 (CI, 1.24–2.76) (44). Moreover, the study showed that the offspring of mothers with hookworm and *M. perstans* infections during pregnancy had significantly higher rates of clinical malaria and asymptomatic parasitemia in comparison with the children of uninfected mothers (45). It was also observed that quarterly albendazole therapy during childhood was associated significantly with the reduced incidence of clinical malaria (see details in Table S1 in Supplementary Material) (46). Henceforth, concurrent of helminth infections and pregnancy have serious consequences on mothers and their offspring; including increase susceptibility, severity, and the risk of mother-to-child transmission of intracellular pathogens, as well as immune response impairment to postnatal immunization.

## Helminth Infections, Malnourishment and Immune Response during Pregnancy: Another Complication of Pregnant Women with Helminth Infections

It should be noted that most of the studies described above are coming from developing countries with poor nutrition status. Without adequate nutrition, immune system fails to produce an effective response to infections (49). On the other hand, helminth infections are more prevalent in developing countries with poor sanitation. Conversely, helminth infections are a cause of malnutrition and immune system impairment (50). In this complex situation, pregnant women with helminth infections may be more susceptible to acquiring of congenital infections due to the synergic immunoregulatory effects of malnutrition, pregnancy, and helminth infections.

As described above, pregnant women with chronic helminth infections are more susceptible to the acquisition and subsequent transmission of malaria (20, 44–48) and HIV (36–38) infections to their offspring. It is also evident that helminth infection associations during pregnancy suppress immune responses to postnatal immunization (40–43).

In conclusion, the synergic immunoregulatory effects of pregnancy and chronic helminth infections offer a new insight into the role of immune response to intracellular pathogens. Therefore, increased susceptibility to several congenital infectious diseases – and their severity – are plausible. Hence, diagnosis and treatment of helminth infections in pregnant women should be given greater consideration.

## Supplementary Material

The Supplementary Material for this article can be found online at http://www.frontiersin.org/Journal/10.3389/fimmu.2014.00053/full
