# Supplementary material for: Are Pregnant Women with Chronic Helminth Infections More Susceptible to Congenital Infections?
Source: Front Immunol. 2014 Feb 12;5:53. doi: 10.3389/fimmu.2014.00053 (PMC3921675; doi:10.3389/fimmu.2014.00053)
Supplement: Supplementary file 1 [file 75215_Abdoli_DataSheet1.DOCX]

**Supplementary table 1**. Risk of congenital infections in pregnant women with chronic helminth infections.

| Study subject and setting | Case, control and matching variables | Main findings | Ref |
| --- | --- | --- | --- |
| To examine the effects of maternal parasitic infections on mother-to-child HIV transmission.  Retrospective cohort study among 936 Kenyan mother and infant pairs between 1996 and 2002. | Cases: HIV positive women (n=83)  Controls: HIV negative women (n=166).  Matched for age, parity, residence, level of education, and date of recruitment to women without HIV. | Significant association between maternal helminth infections and transmission of HIV to infants (*p*= 0.02, OR= 7.3, 95% CL 2.4–33.7).  Significant association between mother-to-child transmission of HIV and higher levels of IL-5/IL-13 in response to helminth antigens (*p* < 0.001).  Vertical transmission of HIV to infants was 1.3 times more in mother co-infected with malaria; but it was not statistically significant (*p*=0.09, OR= 1.2, 95% CL 0.3–5.5). | (1) |
| To determinate the prevalence of malaria–helminth co- infections among HIV-infected pregnant women after 12 months of antiretroviral therapy.  Cross sectional study among 328 HIV-positive pregnant women in Rwanda. | 328 HIV-positive pregnant women (91 cases with helminth infections, 36 cases with malaria infection, 33 cases of malaria- helminth co-infection and 168 cases with non infections). | Significant decrease CD4 count among HIV-positive women with helminth infection (*p*< 0.0005) or malaria–helminth co-infection (*p* < 0.005) than subjects with no infection and subjects with a malaria only infection (*p* < 0.0005 and *p* < 0. 05, respectively).  Significant decrease hemoglobin level among HIV-positive women with helminth infection (*p* < 0.05) or malaria–helminth co-infection (*p* < 0.005) than subjects with no infection. | (2) |
| To determinate the influences of maternal helminth infections and their treatment during pregnancy on infant responses to neonatal BCG immunization.  Randomized, double-blind, placebo-controlled trial of anthelmintic treatment among 104 pregnant women in Uganda. | 104 pregnant women in the second trimester of pregnancy; including: albendazole treated group (n=53) and placebo group (n=51). | Significant decrease maternal IFN-γ responses to CFP of *M. tuberculosis* in women with maternal hookworm infection (*p* = 0.021, OR 0.14, 95% CI 0.02–0.83).  Increase IFN-γ response to CFP in hookworm-infected mothers treated with albendazole (*p* = 0.055).  Significant increase IFN-γ responses to CFP among one-year-old infants of hookworm infected mothers (*p* = 0.013, OR 17.65, 95% CI 1.20–258.66).  Decrease IFN-γ response to CFP in infants of hookworm-infected mothers treated with albendazole (*p*=0.20). | (3) |
| To investigate the influences of maternal helminth infection and their treatment during pregnancy on infant response to BCG and tetanus toxoid.  Randomized, double-blind, placebo-controlled trial of anthelmintic treatment during pregnancy among Ugandan pregnant women. | Infants from helminth infected mothers: analyzed for responses to BCG immunization (n=1506), analyzed for responses to tetanus immunization (n=1433). | Positive association between maternal *Mansonella perstans* infection and infant IL-10 production in response to CFP (aGMR 1.23 95% CI 0.99, 1.50) and TT (aGMR 1.36 95% CI 1.04, 1.80).  Maternal albendazole treatment interacted with both associations (interaction *p*-values=0.02 and 0.001, respectively). | (4) |
| To examine the effects of anthelmintic treatment during pregnancy on infant’s immune response to immunisations at age one year.  Randomized, double-blind, placebo-controlled trial of anthelmintic treatment during pregnancy among Ugandan pregnant women. | 2507 women in the second or third trimester of pregnancy:  628 women treated with albendazole and praziquantel, 625 women treated with albendazole and a praziquantel-matching placebo, 626 women treated with praziquantel and an albendazole-matching placebo and 628 women treated with an albendazole-matching placebo and praziquantel-matching placebo. | Neither albendazole nor praziquantel treatments during pregnancy affected infant’s immune responses to BCG, tetanus, or measles immunisation.  Significant decreases IL-5 (GMR 0.50, 95% CI 0.30–0.81, *p*-values =0·02) and IL-13 (GMR 0.52, 95% CI 0.34–0.82, interaction *p*-values=0.0005) response to tetanus toxoid in infants of mothers with hookworm infection treatment with albendazole. | (5) |
| To evaluate the geographical distribution of *P. falciparum*–helminth co-infection in pregnant women.  Cross-sectional study in a sample of 2507 pregnant women in Uganda. | Prevalence of malaria and helminth co-infection was assessed among pregnant women in Entebbe, Uganda. | The risk of *P. falciparum* infection was 1.53 times higher in mothers infected with hookworm (OR 1.53, 95% CI 1.09 –2.14, *p*=0.014), 2.33 times higher in mothers infected with *M. perstans* (OR 2.33, 95% CI 1.47–3.69, *p*<0.001) and 1.85 times higher in mothers infected both hookworm and *M. perstans* (OR 1.85, 95% CI 1.24–2.76, *p*=0.002). | (6) |
| To examine associations between helminth and malaria infections in pregnancy and malaria in the offspring.  Birth cohort study among 2345 mother–child pairs in Uganda. | Status of maternal helminth and malaria infection determined among 2345 mother during pregnancy, and childhood malaria episodes recorded from birth to age 5 years. | The risk of childhood clinical malaria increased in infants of mothers with hookworm (aHR 1.24, 95% CL, 1.10–1.41) and *M. perstans* infection (aHR 1.20, 95% CI 1.05–1.38). | (7) |
| To examine the impacts of anthelmintic treatment during pregnancy and childhood on immunizations, infections and eczema in childhood.  Randomized, double-blind, placebo-controlled trial of anthelmintic treatment during pregnancy among Ugandan pregnant women. | 2507 pregnant women allocated to receive single-dose albendazole or placebo, and praziquantel or placebo.  2016 of their offspring randomized to receive quarterly single dose albendazole (n=1010), or placebo (n=1006) from age 15 months to 5 years. | Reduce incidence of clinical malaria in children treated with albendazole during childhood (HR 0.85, 95% CI 0.73–0.98, *p* = 0.03).  Increase rate of eczema in the children of mothers who treated with albendazole during pregnancy (HR 1.58, 95% CI 1.15–2.17, *p* = 0.005). | (8) |
| To determinate the prevalence of malaria and intestinal helminth co-infection in pregnant women and its risk factors.  Cross-sectional study in a sample of 746 pregnant women in Ghana. | Prevalence of malaria and intestinal helminth co-infection was assessed in pregnant women presenting for delivery at two hospitals in Ghana. | Of the 746 women, 19.7% (N = 147) positive for *P. falciparum* only, 9.1% (N = 68) positive for helminths only, and 16.6% (N = 124) co-infected.  The risk of *P. falciparum* infection was almost five times higher in women infected with intestinal helminth infections (OR = 4.8, 95% CI = 3.4–40). | (9) |

CFP: crude culture filtrate proteins. BCG: Bacille Calmette-Guérin. aGMR: adjacent Geometric Mean Ratios. TT: tetanus toxoid. aHR: adjusted hazard ratio.

**References:**

1. Gallagher M, Malhotra I, Mungai PL, Wamachi AN, Kioko JM, Ouma JH, et al. The effects of maternal helminth and malaria infections on mother-to-child HIV transmission. *AIDS*. 2005;19(16):1849-55.

2. Ivan E, Crowther N, Rucogoza A, Osuwat L, Munyazesa E, Mutimura E, et al. Malaria and helminthic co-infection among HIV-positive pregnant women: prevalence and effects of antiretroviral therapy. *Acta Trop*. 2012;124:179– 84.

3. Elliott AM, Namujju PB, Mawa PA, Quigley MA, Nampijja M, Nkurunziza PM, et al. ARandomizedcontrolled trial of the effects of albendazole in pregnancy on maternal responses to mycobacterial antigens and infant responses to Bacille Calmette-Guerin (BCG) immunisation [ISRCTN32849447]. *BMC Infect Dis*. 2005;5(1):115.

4. Elliott AM, Mawa PA, Webb EL, Nampijja M, Lyadda N, Bukusuba J, et al. Effects of maternal and infant co-infections, and of maternal immunisation, on the infant response to BCG and tetanus immunisation. *Vaccine*. 2010;29(2):247-55.

5. Hillier SD, Booth M, Muhangi L, Nkurunziza P, Khihembo M, Kakande M, et al. *Plasmodium falciparum* and helminth coinfection in a semiurban population of pregnant women in Uganda. *J Infect Dis*. 2008;198(6):920-7.

6. Ndibazza J, Webb EL, Lule S, Harriet M, Akello M, Oduru G, et al. Associations between maternal helminth and malaria infections in pregnancy, and clinical malaria in the offspring: a birth cohort in Entebbe, Uganda. *J Infect Dis*. 2013;_(_):jit397.

7. Ndibazza J, Mpairwe H, Webb EL, Mawa PA, Nampijja M, Muhangi L, et al. Impact of Anthelminthic Treatment in Pregnancy and Childhood on Immunisations, Infections and Eczema in Childhood: ARandomizedControlled Trial. *PloS one*. 2012;7(12):e50325.

8. Yatich NJ, Yi J, Agbenyega T, Turpin A, Rayner JC, Stiles JK, et al. Malaria and intestinal helminth co-infection among pregnant women in Ghana: prevalence and risk factors. *Am J Trop Med Hyg*. 2009;80(6):896-901.

9. Thigpen MC, Filler SJ, Kazembe PN, Parise ME, Macheso A, Campbell CH, et al. Associations between peripheral *Plasmodium falciparum* malaria parasitemia, human immunodeficiency virus, and concurrent helminthic infection among pregnant women in Malawi. *Am J Trop Med Hyg*. 2011;84(3):379–85.
